# Supplementary material for: U-shaped association between serum triglyceride levels and mortality among septic patients: An analysis based on the MIMIC-IV database
Source: PLoS One. 2023 Nov 27;18(11):e0294779. doi: 10.1371/journal.pone.0294779 (PMC10681221; doi:10.1371/journal.pone.0294779)
Supplement: S1 Table — (DOCX) [file pone.0294779.s001.docx]

**Supplementary Table 1 Characteristics of survivors and non-survivors within ICU.**

| **Variables** | **Total**  **(n=2782)** | **Survivors**  **(n=2295)** | **Non-survivors**  **(n=487)** | ***p* value** |
| --- | --- | --- | --- | --- |
| Age (years) | 63.9 (51.8, 74.5) | 63.2 (55.9, 77.3) | 67.2 (55.9, 77.3) | <0.001 |
| Female (n (%)) | 1119 (40.2) | 935 (40.7) | 184 (37.8) | 0.227 |
| BMI (kg/m^2^) | 28.4 (24.4, 33.8) | 24.3 (24.3, 33.6) | 28.7 (24.5, 34.0) | <0.001 |
| SOFA score | 8.0 (5.0, 12.0) | 7.00 (5.0, 11.0) | 11.0 (7.0, 14.0) | <0.001 |
| **Comorbidities** | | | | |
| Hypertension (n (%)) | 1075 (38.6) | 899 (39.2) | 176 (36.1) | 0.212 |
| Diabetes (n (%)) | 834 (30.0) | 681 (29.7) | 153 (31.4) | 0.446 |
| Hyperlipidemia (n (%)) | 890 (32.0) | 726 (31.6) | 164 (33.7) | 0.380 |
| CPD (n (%)) | 754 (27.1) | 600 (26.1) | 154 (31.6) | 0.013 |
| MI (n (%)) | 516 (18.6) | 418 (18.2) | 98 (20.1) | 0.352 |
| CHF (n (%)) | 902 (32.4) | 715 (31.2) | 187 (38.4) | 0.002 |
| Atherosclerosis (n (%)) | 336 (32.4) | 271 (11.8) | 65 (13.4) | 0.344 |
| Vascular disease (n (%)) | 978 (35.2) | 805 (35.1) | 173 (35.5) | 0.851 |
| Liver disease (n (%)) | 600 (21.6) | 455 (19.8) | 145 (29.8) | <0.001 |
| Renal disease (n (%)) | 593 (21.3) | 463 (20.2) | 130 (26.7) | 0.001 |
| Hypothyroidism (n (%)) | 348 (12.5) | 284 (12.4) | 64 (13.1) | 0.642 |
| Pancreatitis (n (%)) | 202 (7.3) | 175 (7.6) | 27 (5.5) | 0.108 |
| Tumor (n (%)) | 332 (11.9) | 252 (11.0) | 80 (16.4) | 0.001 |
| **During the first 24 hours after ICU admission** | | | | |
| Heart rate (beat/min) | 109.0 (95.0, 124.0) | 108.0 (95.0, 124.0) | 111.0 (96.0, 126.0) | 0.070 |
| MAP (mmHg) | 59.0 (53.00, 66.0) | 59.0 (53.0, 66.0) | 58.0 (52.0, 63.0) | <0.001 |
| Blood glucose (mg/dL) | 167.0 (131.0, 225.0) | 165.0 (130.0, 222.0) | 177.0 (137.0, 241.0) | 0.001 |
| Lactate (mmol/L) | 2.2 (1.6, 3.1) | 2.2 (1.5, 2.9) | 2.2 (1.9, 4.5) | <0.001 |
| WBC (×10^9^/L) | 13.9 (10.1, 19.0) | 13.7 (9.9, 18.6) | 15.5 (11.2, 21.4) | <0.001 |
| Platelet (×10^9^/L) | 209.0 (148.0, 281.0) | 212.0 (151.0, 285.0) | 197.0 (132.0, 267.0) | 0.001 |
| Hematocrit (%) | 35.5 (30.5, 40.9) | 35.6 (30.7, 41.0) | 34.5 (29.3, 40.5) | 0.005 |
| Hemoglobin (g/L) | 11.60 (9.90, 13.5) | 11.7 (10.0, 13.5) | 11.1 (9.5, 13.2) | 0.001 |
| BUN (mg/dL) | 24.0 (16.0, 39.0) | 22.0 (15.0, 36.0) | 30.0 (20.0, 51.0) | <0.001 |
| Creatinine (mg/dL) | 1.2 (0.9, 2.0) | 1.2 (0.8, 1.9) | 1.5 (1.0, 2.4) | <0.001 |
| ALT (IU/L) | 31.0 (21.0, 50.0) | 31.0 (21.0, 49.0) | 31.0 (21.0, 59.0) | 0.099 |
| AST (IU/L) | 47.0 (32.0, 79.0) | 47.0 (31.0, 73.0) | 47.0 (37.0, 104.0) | <0.001 |
| Albumin (g/dL) | 3.3 (3.0, 3.5) | 3.3 (3.1, 3.5) | 3.3 (2.8, 3.4) | 0.001 |
| Total bilirubin (mg/dL) | 0.8 (0.5, 1.4) | 0.8 (0.5, 1.2) | 0.8 (0.6, 2.2) | <0.001 |
| RRT within 7 days (n (%)) | 443 (15.9) | 298 (13.0) | 145 (29.8) | <0.001 |
| TG_max_ level (mg/dL) | 145.0 (93.0, 249.0) | 147.0 (94.0, 250.0) | 141.0 (89.0, 238.0) | 0.188 |
| TG_min_ level (mg/dL) | 132.5 (88.0, 207.0) | 134.0 (89.0, 211.0) | 126.0 (84.0, 191.0) | 0.008 |

**Note:** Continuous variables (age, BMI, MAP, *et al.*) were presented as median (IQR). Categorical variables (hypertension, CPD, MI, *et al.*) were presented as frequencies (percentages). The differences between survivors and non-survivors were analyzed by Mann-Whitney U test, and Chi-square. BMI: body mass index; SOFA: sequential organ failure assessment; CPD: chronic pulmonary disease; MI: myocardial infarct; CHF: congestive heart failure; MAP: mean arterial pressure; WBC: white blood cell; BUN: blood urea nitrogen; ALT: alanine transaminase; AST: aspartate aminotransferase; RRT: renal replacement therapy; TG_max_: maximum value of triglycerides; TG_min_: minimum value of triglycerides.
