# Supplementary material for: U-shaped association between serum triglyceride levels and mortality among septic patients: An analysis based on the MIMIC-IV database
Source: PLoS One. 2023 Nov 27;18(11):e0294779. doi: 10.1371/journal.pone.0294779 (PMC10681221; doi:10.1371/journal.pone.0294779)
Supplement: S2 Table — (DOCX) [file pone.0294779.s002.docx]

**Supplementary Table 2 Characteristics of survivors and non-survivors in hospital.**

| **Variables** | **Total**  **(n=2782)** | **Survivors**  **(n=2131)** | **Non-survivors**  **(n=651)** | ***p* value** |
| --- | --- | --- | --- | --- |
| Age (years) | 63.9 (51.8, 74.5) | 62.61 (50.2, 73.0) | 67.5 (55.9, 77.9) | <0.001 |
| Female (n (%)) | 1119 (40.22) | 869 (40.8) | 250 (38.4) | 0.279 |
| BMI (kg/m^2^) | 28.4 (24.4, 33.8) | 28.4 (24.5, 33.8) | 28.2 (24.1, 33.8) | <0.001 |
| SOFA score | 8.0 (5.0, 12.0) | 7.0 (5.0, 11.0) | 10.0 (7.0, 13.0) | <0.001 |
| **Comorbidities** | | | | |
| Hypertension (n (%)) | 1075 (38.6) | 846 (39.7) | 229 (35.2) | 0.038 |
| Diabetes (n (%)) | 834 (30.0) | 635 (29.8) | 199 (30.6) | 0.851 |
| Hyperlipidemia (n (%)) | 890 (32.0) | 684 (32.1) | 206 (31.6) | 0.828 |
| CPD (n (%)) | 754 (27.1) | 567 (26.6) | 187 (28.7) | 0.287 |
| MI (n (%)) | 516 (18.6) | 385 (18.1) | 131 (20.1) | 0.237 |
| CHF (n (%)) | 902 (32.4) | 651 (30.6) | 251 (38.6) | <0.001 |
| Atherosclerosis (n (%)) | 336 (32.4) | 245 (30.6) | 91 (38. 6) | 0.018 |
| Vascular disease (n (%)) | 978 (35.2) | 736 (34.5) | 242 (37.2) | 0.218 |
| Liver disease (n (%)) | 600 (21.6) | 405 (19.0) | 195 (30.0) | <0.001 |
| Renal disease (n (%)) | 593 (21.3) | 428 (20.1) | 165 (25.4) | 0.004 |
| Hypothyroidism (n (%)) | 348(12.5) | 263 (12.3) | 85 (13.1) | 0.629 |
| Pancreatitis (n (%)) | 202(7.3) | 168 (17.9) | 34 (5.2) | 0.022 |
| Tumor (n (%)) | 332 (11.9) | 218 (10.2) | 114 (17.5) | <0.001 |
| **During the first 24 hours after ICU admission** | | | | |
| Heart rate (beat/min) | 109.0 (95.0, 124.0) | 108.0 (95.0, 124.0) | 111.0 (96.0, 126.0) | 0.054 |
| MAP (mmHg) | 59.0 (53.00, 66.0) | 59.0 (53.0, 66.0) | 58.0 (52.0, 64.0) | <0.001 |
| Blood glucose (mg/dL) | 167.0 (131.0, 225.0) | 165.0(130.0, 223.0) | 173.0 (137.0, 231.0) | 0.011 |
| Lactate (mmol/L) | 2.2 (1.6, 3.1) | 2.2 (1.5, 2.9) | 2.2 (1.9, 4.3) | <0.001 |
| WBC (×10^9^/L) | 13.9 (10.1, 19.0) | 13.6 (9.9, 18.4) | 15.2 (10.8) | <0.001 |
| Platelet (×10^9^/L) | 209.0 (148.0, 281.0) | 212.0 (153.0, 286.0) | 196.0 (127.0, 270.0) | <0.001 |
| Hematocrit (%) | 35.5 (30.5, 40.9) | 35.9 (31.0, 41.0) | 34.0 (28.9, 40.2) | <0.001 |
| Hemoglobin (g/L) | 11.60 (9.90, 13.5) | 11.7 (10.0, 13.5) | 11.0 (9.3, 13.1) | <0.001 |
| BUN (mg/dL) | 24.0 (16.0, 39.0) | 22.0 (15.0, 36.0) | 28.0 (19.0, 49.0) | <0.001 |
| Creatinine (mg/dL) | 1.2 (0.9, 2.0) | 1.2 (0.8, 1.9) | 1.4 (1.0, 2.3) | <0.001 |
| ALT (IU/L) | 31.0 (21.0, 50.0) | 31.0 (21.0, 49.0) | 31.0 (21.0, 56.0) | 0.124 |
| AST (IU/L) | 47.0 (32.0, 79.0) | 47.0 (31.0, 73.0) | 47.0 (37.0, 101.0) | <0.001 |
| Albumin (g/dL) | 3.3 (3.0, 3.5) | 3.3 (3.1, 3.6) | 3.3 (2.8, 3.4) | <0.001 |
| Total bilirubin (mg/dL) | 0.8 (0.5, 1.4) | 0.8 (0.5, 1.2) | 0.8 (0.6, 2.2) | <0.001 |
| RRT within 7 days (n (%)) | 443 (15.9) | 275 (12.9) | 168 (25.8) | <0.001 |
| TG_max_ level (mg/dL) | 145.0 (93.0, 249.0) | 148.0 (95.0, 253.0) | 137.0 (88.0, 231.0) | 0.030 |
| TG_min_ level (mg/dL) | 132.5 (88.0, 207.0) | 135.0 (90.0, 211.0) | 123.0 (84.0, 192.0) | 0.001 |

**Note:** Continuous variables (age, BMI, MAP, *et al.*) were presented as median (IQR). Categorical variables (hypertension, CPD, MI, *et al.*) were presented as frequencies (percentages). The differences between survivors and non-survivors were analyzed by Mann-Whitney U test, and Chi-square. BMI: body mass index; SOFA: sequential organ failure assessment; CPD: chronic pulmonary disease; MI: myocardial infarct; CHF: congestive heart failure; MAP: mean arterial pressure; WBC: white blood cell; BUN: blood urea nitrogen; ALT: alanine transaminase; AST: aspartate aminotransferase; RRT: renal replacement therapy; TG_max_: maximum value of triglycerides; TG_min_: minimum value of triglycerides
