# Supplementary material for: U-shaped association between serum triglyceride levels and mortality among septic patients: An analysis based on the MIMIC-IV database
Source: PLoS One. 2023 Nov 27;18(11):e0294779. doi: 10.1371/journal.pone.0294779 (PMC10681221; doi:10.1371/journal.pone.0294779)
Supplement: S3 Table — (DOCX) [file pone.0294779.s003.docx]

**Supplementary Table 3 Mortality of different serum TG_max_ groups (clinical boundary values).**

| **Variables** | **Total** | **＜150 mg/dL** | **150-500 mg/dL** | ***p* value** |
| --- | --- | --- | --- | --- |
| 28-day mortality (n(%)) | 507(22.32) | 340 (23.5) | 167 (20.2) | 0.063 |
| ICU mortality (n(%)) | 401(17.65) | 270 (18.7) | 131 (15.8) | 0.083 |
| In-hospital mortality (n(%)) | 543(23.90) | 363 (25.1) | 180 (21.7) | 0.067 |

**Note:** Data were presented as frequencies (percentages). The differences between the 2 TG_max_ groups were analyzed by Chi-square. TG_max_: maximum value of triglycerides; ICU: Intensive Care Unit.
