# Supplementary figures and images for: U-shaped association between serum triglyceride levels and mortality among septic patients: An analysis based on the MIMIC-IV database
Source: PLoS One. 2023 Nov 27;18(11):e0294779. doi: 10.1371/journal.pone.0294779 (PMC10681221; doi:10.1371/journal.pone.0294779)

Supplementary Figure 1 Number of participants with missing data for each variable of interest.


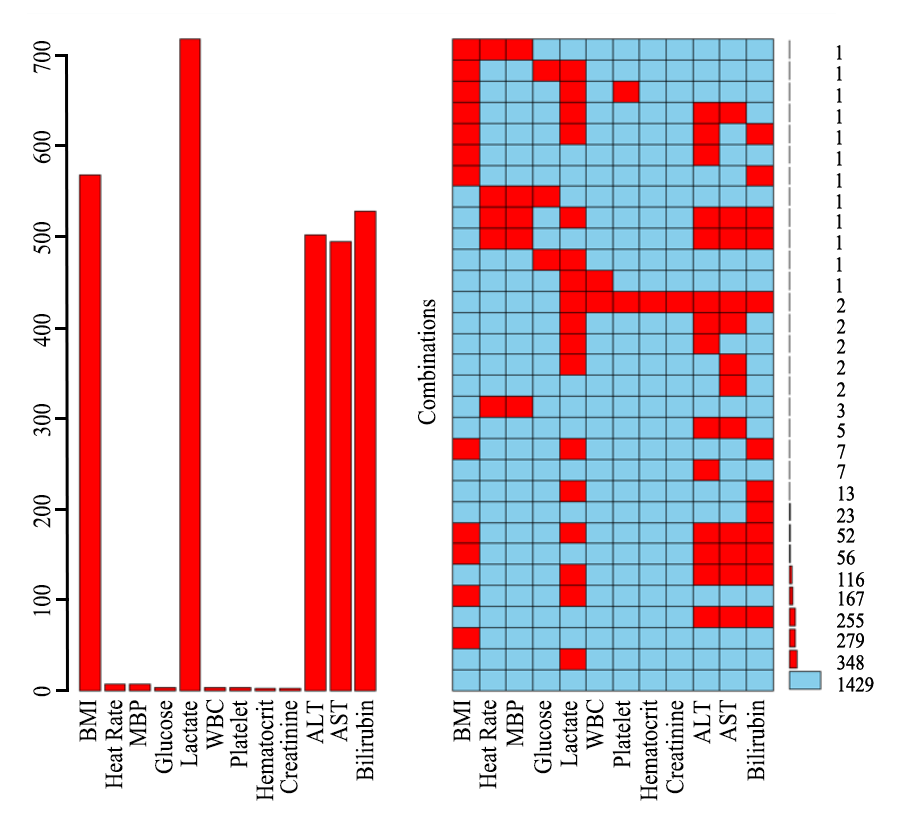

Supplement: S1 Fig — (DOCX) [file pone.0294779.s004.docx]
